# Neutral ceramidase deficiency protects against cisplatin-induced acute kidney injury

**DOI:** 10.1016/j.jlr.2022.100179

**Published:** 2022-02-10

**Authors:** Sophia M. Sears, Tess V. Dupre, Parag P. Shah, Deanna L. Davis, Mark A. Doll, Cierra N. Sharp, Alexis A. Vega, Judit Megyesi, Levi J. Beverly, Ashley J. Snider, Lina M. Obeid, Yusuf A. Hannun, Leah J. Siskind

**Affiliations:** 1 Department of Pharmacology & Toxicology, University of Louisville, Louisville, KY, USA; 2 Department of Medicine, University of Louisville, Louisville, KY, USA; 3 James Graham Brown Cancer Center, University of Louisville, Louisville, KY, USA; 4 Department of Biochemistry & Molecular Genetics, University of Louisville, Louisville, KY, USA; 5 Division of Nephrology, Department of Internal Medicine, University of Arkansas for Medical Sciences and Central Arkansas, Veterans Healthcare System, Little Rock, AR, USA; 6 Department of Nutritional Sciences, College of Agriculture and Life Sciences, University of Arizona, Tucson, AZ, USA; 7 Department of Medicine, Stony Brook University, Stony Brook, NY, USA; 8 Stony Brook Cancer Center, Stony Brook University, Stony Brook, NY, USA; 9 Northport Veteran Affairs Medical Center, Northport, NY, USA

**Keywords:** autophagy, ceramide, sphingosine-1-phosphate, renal disease, animal models, cisplatin, sphingosine metabolism, chemotherapy, ER stress, chloroquine, AKI, acute kidney injury, *B2m*, beta-2-microglobulin, BUN, blood urea nitrogen, CC3, cleaved caspase 3, CHOP, C/EBP homologous protein, CKD, chronic kidney disease, CQ, chloroquine, *Cxcl1*, chemokine (C-X-C Motif) ligand 1, ERK, extracellular receptor kinase, *Il-6*, interleukin-6, IRE1α, inositol requiring enzyme-1 alpha, JNK, c-jun n-terminal kinase, LC3B, microtubule associated protein light chain 3, *Mcp-1*, monocyte chemoattractant protein-1, nCDase, neutral ceramidase, NGAL, neutrophil gelatinase-associated lipocalin, p62, sequestosome 1/p62, PCNA, proliferating cell nuclear antigen, p-eIF2α, phosphorylated- eukaryotic initiation factor 2 alpha, p-ERK, phosphorylated-extracellular receptor kinase, p-JNK, phosphorylated-c-jun n-terminal kinase, S1P, sphingosine-1-phosphate, S1PR1, S1P receptor 1, SCr, serum creatinine, *Tnfα*, tumor necrosis factor alpha

## Abstract

Cisplatin is a commonly used chemotherapeutic for the treatment of many solid organ cancers; however, its effectiveness is limited by the development of acute kidney injury (AKI) in 30% of patients. AKI is driven by proximal tubule cell death, leading to rapid decline in renal function. It has previously been shown that sphingolipid metabolism plays a role in regulating many of the biological processes involved in cisplatin-induced AKI. For example, neutral ceramidase (nCDase) is an enzyme responsible for converting ceramide into sphingosine, which is then phosphorylated to become sphingosine-1-phosphate, and our lab previously demonstrated that nCDase knockout (nCDase−/−) in mouse embryonic fibroblasts led to resistance to nutrient and energy deprivation–induced cell death via upregulation of autophagic flux. In this study, we further characterized the role of nCDase in AKI by demonstrating that nCDase−/− mice are resistant to cisplatin-induced AKI. nCDase−/− mice display improved kidney function, reduced injury and structural damage, lower rates of apoptosis, and less ER stress compared to wild-type mice following cisplatin treatment. Although the mechanism of protection is still unknown, we propose that it could be mediated by increased autophagy, as chloroquine treatment resensitized nCDase−/− mice to AKI development. Taken together, we conclude that nCDase may represent a novel target to prevent cisplatin-induced nephrotoxicity.

Cisplatin (*cis*-diamminedichloridoplatinum(II)) is an effective chemotherapeutic for the treatment of many solid organ cancers (1, 2, 3). Unfortunately, the success of cisplatin in treating these cancers is limited by its nephrotoxicity. Thirty percent of patients treated with cisplatin develop cisplatin-induced acute kidney injury (AKI) (4, 5, 6, 7). AKI is defined as a rapid decline in glomerular filtration rate, clinically measured by increases in blood urea nitrogen (BUN) or serum creatinine (SCr) (8). AKI is primarily mediated by renal proximal tubule cell death, inflammation, and impaired microvasculature (9). Development of AKI not only limits the ability to treat cancer patients but also puts patients at risk for long-term renal effects (8, 10, 11). As development of cisplatin-induced AKI is thought to be largely driven by cell death (4, 6), protecting proximal tubule cells from death is an attractive strategy to prevent kidney structural damage and maintain function. Sphingolipid metabolism in the kidney is recognized as a regulator of several cellular processes, including cell death, that play a role in the development of AKI and other renal diseases (12, 13, 14); thus, this connection has been explored by our lab.

Sphingolipids are a class of bioactive lipids with a common sphingoid base backbone. Sphingolipid metabolism centers around the formation and breakdown of ceramides (15, 16). Neutral ceramidase (nCDase) is an enzyme involved in sphingolipid metabolism that cleaves ceramide into sphingosine, which is then recycled back to ceramide or phosphorylated to become sphingosine-1-phosphate (S1P) (17, 18, 19, 20). The balance of these three bioactive lipids is regulated by dynamic processes and is thought to play an important role in regulating cellular stress responses (15, 16). In particular, ceramide, sphingosine, and S1P have been implicated in regulation of cell death and autophagy (16). Manipulating the balance of ceramide has also been shown to play a role in cisplatin-induced AKI. Inhibition of ceramide generation protected mice from cisplatin-induced AKI, while inhibition of glucosylceramide synthase, an enzyme that glycosylates ceramide species to generate glycosphingolipids, exacerbated cisplatin-induced AKI (21).

Our lab demonstrated that nCDase knockout (nCDase^−/−^) in mouse embryonic fibroblasts protected cells from nutrient and energy deprivation–induced cell death (22). This protection was mediated via upregulation of autophagy and mitophagy (22). Autophagy is known to play a protective role in cisplatin-induced AKI by decreasing levels of apoptotic cell death (23, 24). Additionally, nCDase^−/−^ mice were found to be protected in a model of traumatic brain injury due to preservation of mitochondrial function (25). Mitochondrial dysfunction is also known to be a major mediator of cisplatin-induced AKI (4). Therefore, we hypothesized that nCDase deficiency would prevent development of cisplatin-induced AKI via upregulation of autophagy and inhibition of cell death.

In this study, we utilized wild-type and nCDase^−/−^ C57BL/6 mice in the cisplatin-induced AKI model. Our data demonstrate that loss of nCDase attenuates AKI development following 20 mg/kg cisplatin treatment as evidenced by markers of kidney function, kidney injury, cell death, and kidney pathology. Furthermore, we demonstrate that chloroquine (CQ) treatment exacerbates development of AKI in nCDase^−/−^ mice. This suggests that nCDase deficiency could be mediating protection from cisplatin-induced AKI by increasing basal autophagy and allowing cells to survive cisplatin-induced injury. These data indicate that nCDase may be a feasible target for prevention of cisplatin nephrotoxicity.

## Materials and methods

### Animals

nCDase^−/−^ mice were generated in the laboratory of Dr Richard L. Proia (NIDDK, National Institutes of Health) (26). These mice were backcrossed for 10 generations onto a C57BL/6 background (27). nCDase^−/−^ knockout mice were bred in-house, and all experiments were performed at 8 weeks of age. Wild-type mice (C57BL/6, male, 8 weeks old) were either bred in-house or purchased from The Jackson Laboratory (Bar Harbor, ME) and allowed to acclimate for 4 weeks prior to initiation of experiments. All mice were maintained on a 12-h light–dark cycle and provided food and water ad libitum. All animal procedures were approved by the Institutional Animal Care and Use Committee and followed the guidelines of the American Veterinary Medical Association. Pharmacy grade cisplatin (1 mg/ml) from either Teva or Intas Pharmaceuticals was obtained from the University of Louisville Hospital pharmacy. Cisplatin 20 mg/kg (in 0.9% normal saline) was administered by intraperitoneal injection at time zero. All animals were injected with cisplatin at the same time of the day as there are circadian influences on the response of the kidney to cisplatin (28, 29). CQ diphosphate salt (Sigma-Aldrich, C6628) was dissolved in 0.9% normal saline into a 15 mg/ml stock solution. Mice were intraperitoneally injected with CQ at 60 mg/kg in 0.9% normal saline 1 h before cisplatin dosing and 24 and 48 h after dosing. Euthanasia was performed 72 h after cisplatin dosing. Blood was collected and plasma was prepared and frozen at -80°C; urine and kidneys were collected, flash frozen in liquid nitrogen, and stored at -80°C until use; one kidney was sliced and fixed in 10% neutral-buffered formalin.

### Assessment of kidney function and injury markers

BUN (AMS Diagnostics, 40146) and SCr (Point Scientific Inc, C7548-120) levels were determined on plasma samples using kits, following the manufacturers’ instructions. ELISAs for neutrophil gelatinase-associated lipocalin (NGAL) (R&D Systems, DY1857) were performed on the urine as directed by the manufacturer and as previously described (30).

### Protein quantification and Western blot analysis

Homogenates were made from kidney cortex using cell extraction buffer (Thermo Fisher Scientific, FNN0011), containing a cOmplete protease inhibitor cocktail tablet (Roche, 4693159001) and PhosSTOP phosphatase inhibitor cocktail tablet (Roche, 4906837001). Protein concentrations were determined using Bradford Reagent (Bio-Rad, 5000001). Kidney homogenate (40 μg) was separated on 4%–12% gradient Tris–Glycine–SDS polyacrylamide gels, transferred to PVDF membranes, and proteins were detected by chemiluminescence substrate. Antibodies were purchased from Cell Signaling unless otherwise noted: inositol requiring enzyme-1α, #3294; extracellular receptor kinase (ERK), #4695; phosphorylated-ERK(p-ERK), #4370; cleaved caspase 3 (CC3), #9664; C/EBP homologous protein, #2895; c-jun n-terminal kinase (JNK), #9258; phosphorylated-JNK (p-JNK), #4668; proliferating cell nuclear antigen (PCNA), #13110; phosphorylated-eukaryotic initiation factor 2α, #3398; sequestosome 1/p62 (p62), #5114; microtubule-associated protein light chain 3, #3868; α-tubulin (Santa Cruz Biotechnology, sc-5286) and β-actin (Sigma-Aldrich, A2228).

### Gene expression

RNA was isolated using TRIzol (Thermo Fisher Scientific, 15596026) or E.Z.N.A. Total RNA Kit (Omega, R6834-02) per the manufacturers’ protocol. Complementary DNA was synthesized from 1 μg RNA with High-Capacity cDNA Reverse Transcriptase PCR (Life Technologies, 4368814) per the manufacturer’s instructions. The following TAQ-man assays (Life Technologies) were used: tumor necrosis factor α (*Tnfα*, Mm00443258_m1), interleukin-6 (*Il-6*, Mm00446190_m1), chemokine (C-X-C Motif) ligand 1 (*Cxcl1*, Mm04207460_m1), monocyte chemoattractant protein-1 (*Mcp-1*, Mm00-441242_m1), and the housekeeping gene beta-2-microglobulin (*B2m,* Mm00437762_m1). The primer for kidney injury molecule-1 was self-designed with the following sequences: forward AGATCCACACATGTACCAACATCAA and reverse CAGTGCCATTCCAGTCTGGTTT. Real-time quantitative RT-PCR was done with either iTaq Universal Probes Supermix (Bio-Rad, 172-5134) or iTaq Universal Sybr Green Supermix (Bio-Rad, 172-5124).

### Histology

Kidney histology and immunohistochemistry were done as previously described (30, 31, 32). Briefly, kidney sections (5 μm) were stained with H&E and periodic acid–Schiff (PAS), and the degree of morphologic changes was determined by light microscopy in a blinded fashion. The following measures were chosen as an indication of morphologic damage to the kidney after drug treatment: proximal tubule degradation, loss of brush border, tubular casts, proximal tubule dilation, and proximal tubule necrosis. These measures were evaluated on a scale from 0 to 4, which ranged from not present (0), mild (1), moderate (2), severe (3), and very severe (4).

### TUNEL staining

ApopTag Red In Situ Apoptosis Detection Kit (Millipore, S7165) was used for immunofluorescent detection of apoptotic cells following the manufacturer’s protocol. ApopTag Plus Peroxidase In Situ Apoptosis Kit (Millipore, S7101) was used for bright-field detection of apoptotic cells following the manufacturer’s protocol.

### Statistical analysis

Data are expressed as means ± SEM for all experiments. Multiple comparisons of normally distributed data were analyzed by two-way ANOVA, as appropriate, and group means were compared using Tukey post-tests. The criterion for statistical differences was *P* < 0.05 for all comparisons.

## Results

### nCDase^−/−^ mice are resistant to cisplatin-induced AKI

**Fig. 1:**
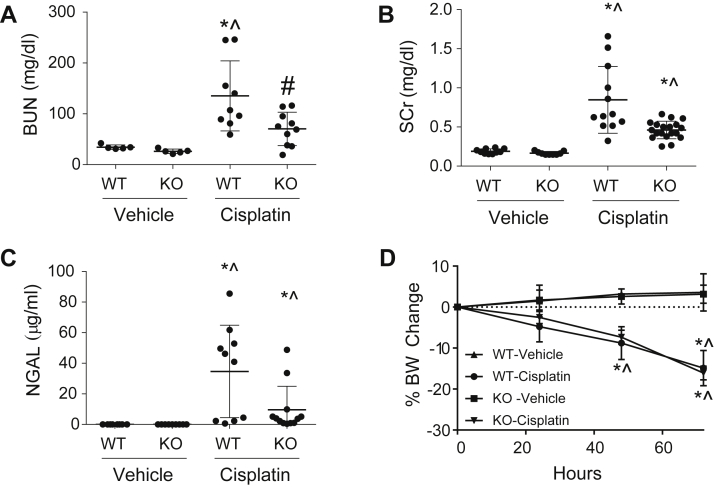
Loss of neutral ceramidase (nCDase) attenuates cisplatin-induced acute kidney injury (AKI). Markers of kidney function: (A) blood urea nitrogen (BUN) and (B) serum creatinine (SCr) were measured from plasma 72 h following administration of cisplatin (20 mg/kg, i.p.) or vehicle. C: Urinary neutrophil gelatinase-associated lipocalin (NGAL) was assessed 72 h following cisplatin administration. D: Percent body weight (BW) change from baseline was monitored each day after injection. WT indicates wild-type mice and KO indicates nCDase^−/−^ mice. Statistical differences were determined by a two-way ANOVA followed by a Tukey post-test. ∗Statistically different than vehicle-treated WT mice. #Statistically different than cisplatin-treated WT mice. ˆStatistically different than vehicle-treated KO mice. Data expressed as mean ± SEM, n = 5–10.

Seventy-two hours post treatment with 20 mg/kg cisplatin, nCDase^−/−^ mice demonstrated improved renal function with significantly reduced BUN (Fig. 1A) and lower levels of SCr (Fig. 1B) than wild-type cisplatin-treated mice. Kidney injury, measured by urinary NGAL, was also decreased in nCDase^−/−^ mice (Fig. 1C). Overt toxicity, as indicated by greater than 10% body weight loss, was comparable between wild-type and nCDase^−/−^ mice (Fig. 1D).

**Fig. 2:**
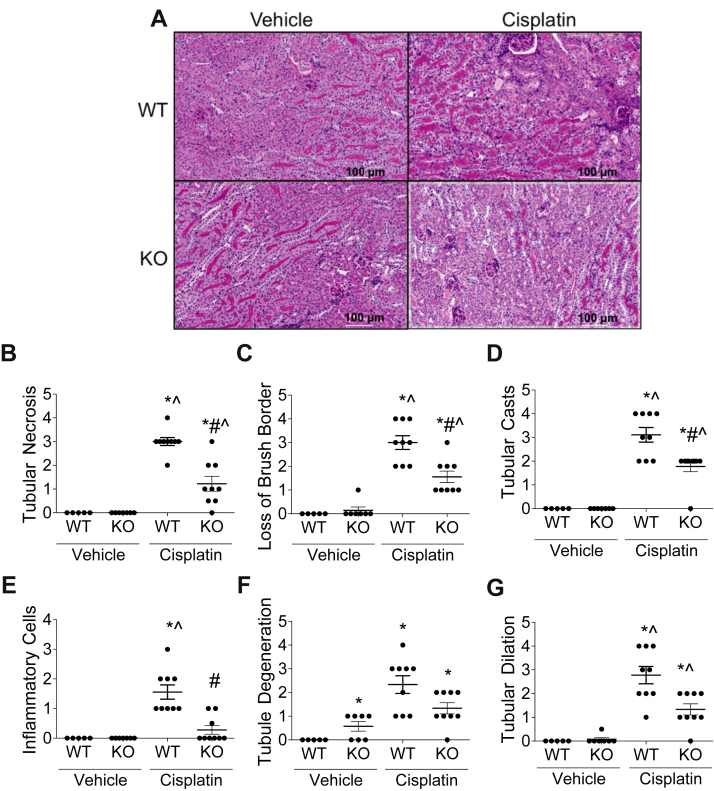
Loss of neutral ceramidase (nCDase) protects from cisplatin-induced deterioration in kidney pathology. Mice were euthanized 72 h following 20 mg/kg intraperitoneal cisplatin or vehicle treatment. Renal histological changes were assessed on 5-μm-thick paraffin-embedded, H&E and PAS-stained sections. A: Representative images of renal histology. B: Tubular necrosis, (C) loss of proximal tubule brush borders, (D) proximal tubule cast formation, (E) inflammatory cells, (F) tubule degeneration, and (G) tubule dilation were assessed as markers of histological changes. For figures (B–G), scoring of the sections was performed in a blinded manner by renal pathologist Dr Megyesi using a scale of 0–4 (0 = not present, 1 = mild, 2 = moderate, 3 = severe, and 4 = very severe renal histological changes in the proximal tubules). WT indicates wild-type mice and KO indicates nCDase^−/−^ mice. Statistical differences were measured by individual chi-square tests. ∗Statistically different than vehicle-treated WT mice. #Statistically different than cisplatin-treated WT mice. ˆStatistically different than vehicle-treated KO mice. Data expressed as mean ± SEM, n = 5–10.

Cisplatin-induced AKI presents with tubular necrosis, loss of brush border, regeneration, cast formation, degeneration, and dilation (33). H&E-stained and PAS-stained kidney sections of nCDase^−/−^ and wild-type mice were scored by a pathologist in a blinded manner. Histology demonstrated improvement in tissue damage in nCDase^−/−^ mice, with significantly less tubular necrosis, loss of brush border, and tubular cast formation compared with wild-type cisplatin-treated mice (Fig. 2A–D). There was also less tubule degeneration and dilation in nCDase^−/−^ mice, although results were not significant (Fig. 2F, G).

**Fig. 3:**
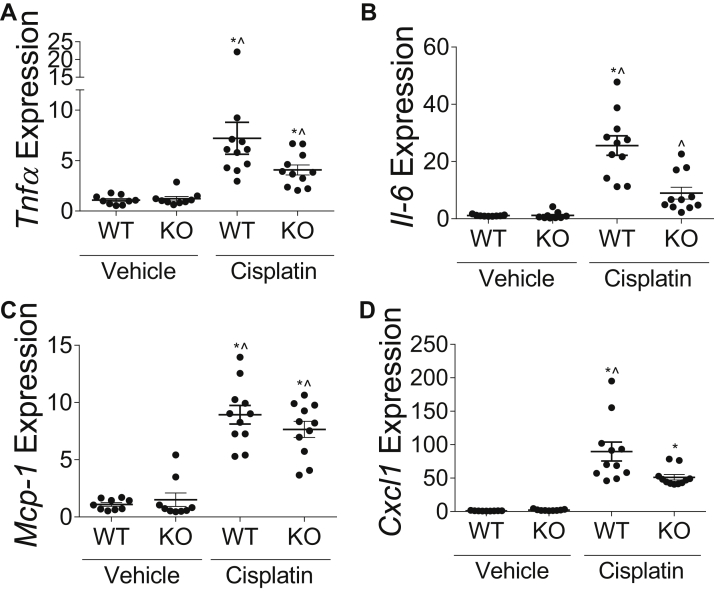
Loss of neutral ceramidase (nCDase) decreases inflammatory cytokine and chemokine production in the kidney following cisplatin treatment. Mice were euthanized 72 h following 20 mg/kg intraperoneal cisplatin or vehicle treatment. mRNA expression relative to control gene beta-2-microglobulin of (A) tumor necrosis factor-α (*Tnfα*), (B) interleukin-6 (*Il-6*), (C) monocyte chemotactic protein-1 (*Mcp-1*), and (D) chemokine (C-X-C) ligand-1 (*Cxcl1*) were measured via real-time qRT-PCR. WT indicates wild-type mice and KO indicates nCDase^−/−^ mice. Statistical differences were determined by two-way ANOVA followed by Tukey post-test. ∗Statistically different than vehicle-treated WT mice. #Statistically different than cisplatin-treated WT mice. ˆStatistically different than vehicle-treated KO mice. Data expressed as mean ± SEM, n = 5–10.

nCDase^−/−^ mice had significantly less inflammatory cells present in the kidney as measured by pathology compared to wild-type mice 72 h post cisplatin treatment (Fig. 2E). This corresponds with lower mRNA expression of inflammatory cytokines *Tnfα*, *Il-6*, *Mcp-1*, and *Cxcl1* in the kidney cortex of cisplatin-treated nCDase^−/−^ mice compared to wild-type cisplatin-treated mice (Fig. 3A–D). Taken together, these results indicate that nCDase^−/−^ mice had improved kidney function, less injury, less structural damage, and less inflammation as compared to wild-type mice 72 h post cisplatin treatment.

### nCDase^−/−^ mice have lower levels of apoptosis and ER stress induction following 20 mg/kg cisplatin treatment

**Fig. 4:**
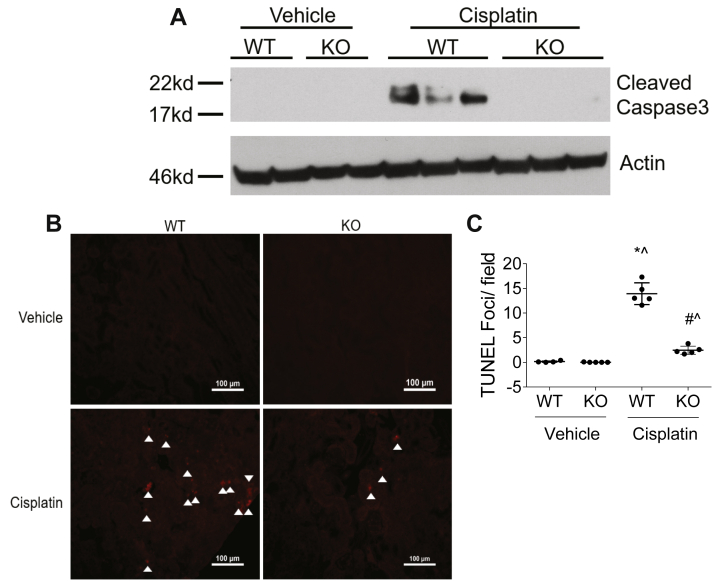
Loss of neutral ceramidase (nCDase) attenuates cisplatin-induced apoptosis. A: Western blot analysis was performed to assess relative protein levels of the indicated proteins in the renal cortex of mice. Samples from the kidney cortex were prepared from mice euthanized 72 h after cisplatin administration. n = 2–3. The same protein lysates and loading control gene were used for Figures 5 and 6A. B, C: TUNEL assays were performed on paraffin-embedded kidney sections as an index of apoptosis. B: Representative photomicrographs of TUNEL assays. C: TUNEL quantification. Statistical differences were measured by two-way ANOVA followed by Tukey post-test. Data expressed as mean ± SEM. n = 5. WT indicates wild-type mice and KO indicates nCDase^−/−^ mice. ∗Statistically different than vehicle-treated WT. #Statistically different than cisplatin-treated WT. ˆStatistically different from vehicle-treated KO.

**Fig. 5:**
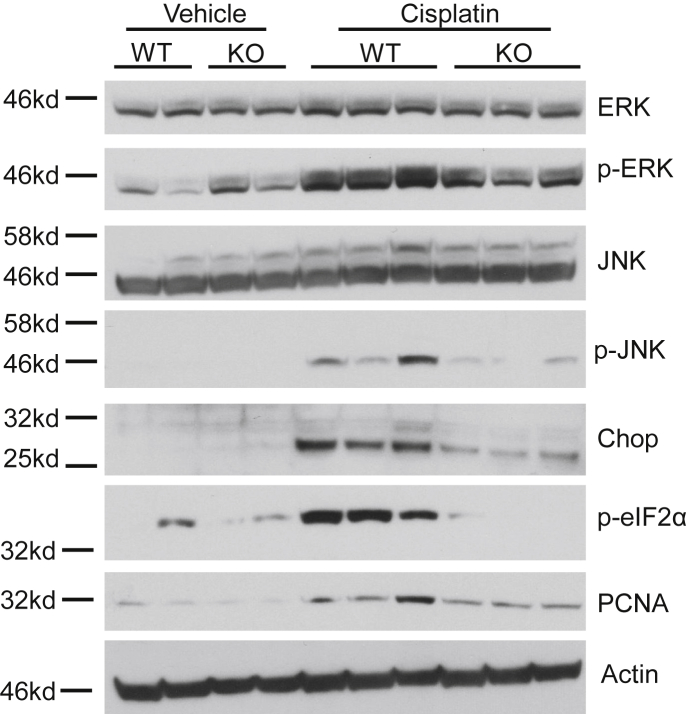
Loss of neutral ceramidase (nCDase) attenuates cisplatin-induced ER stress and proliferation. Western blot analysis was performed to assess relative protein levels of the indicated proteins in the renal cortex of mice. Samples from the kidney cortex were prepared from mice sacrificed 72 h after cisplatin administration. WT indicates wild-type mice and KO indicates nCDase^−/−^ mice. n = 2–3. The same protein lysates and loading control gene were used for Figures 4A and 6A.

A hallmark of cisplatin-induced AKI is induction of cell death. nCDase^−/−^ mice treated with cisplatin had no detectable expression of CC3, a marker of apoptosis, whereas cisplatin-treated wild-type mice had obvious induction of CC3 expression (Fig. 4A). TUNEL staining was also performed to assess DNA breaks formed during apoptosis. nCDase^−/−^ mice had significantly less TUNEL+ cells compared to wild-type mice after cisplatin treatment (Fig. 4B, C). Lastly, PCNA expression was measured to assess the level of cell proliferation occurring in the kidney after cisplatin treatment. nCDase^−/−^ mice had reduced PCNA induction by cisplatin compared to wild-type mice (Fig. 5). These results indicate that nCDase^−/−^ mice are protected from apoptotic cell death and cellular proliferation following 20 mg/kg cisplatin treatment.

Cisplatin is also known to induce ER stress, often leading to cell death (34). ER stress induction was assessed via protein expression of p-ERK, p-JNK, p-eIF2α, and CHOP. Cisplatin-treated nCDase^−/−^ mice had lower expression of these markers compared to cisplatin-treated wild-type mice (Fig. 5). These results suggest that nCDase^−/−^ mice are resistant to ER stress induction in the kidney following 20 mg/kg cisplatin treatment.

### CQ treatment inhibits autophagy in cisplatin-treated nCDase^−/−^ mice

**Fig. 6:**
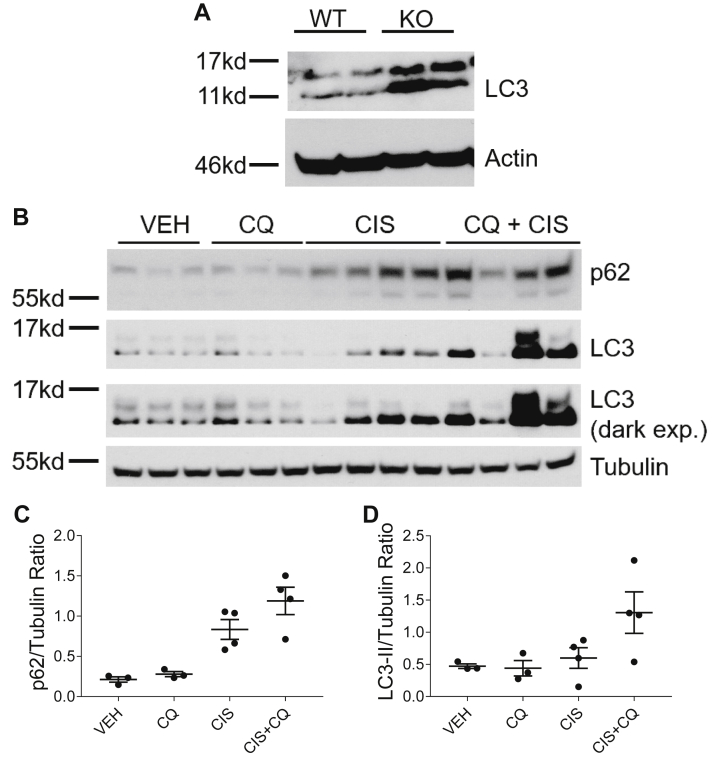
Chloroquine inhibits autophagy induction in cisplatin-treated neutral ceramidase knockout (nCDase^−/−^) mice. A: Western blot analysis was performed to assess relative basal protein levels of the indicated proteins in the renal cortex of nCDase^−/−^ and wild-type mice. n = 2. The same protein lysates and loading control gene were used for Figures 4A and 5. B: Mice were euthanized 72 h following treatment. Western blot analysis was performed to assess relative protein levels of the indicated proteins in the renal cortex of nCDase^−/−^ mice. n = 3–4. VEH indicates vehicle-treated nCDase^−/−^ mice, CQ indicates chloroquine-treated nCDase^−/−^ mice, CIS indicates cisplatin-treated nCDase^−/−^ mice, and CIS+CQ indicates cisplatin and chloroquine–treated nCDase^−/−^ mice. The same protein lysates and loading control gene were used for Figure 9A. C: Quantification of p62 expression relative to tubulin. D: Quantification of LC3II expression relative to tubulin.

We first examined basal expression of LC3 in the kidneys of nCDase^−/−^ and wild-type mice. nCDase^−/−^ mice increased expression of LC3 compared to wild-type mice, suggesting upregulation of autophagy (Fig. 6A). nCDase^−/−^ mice were then administered 60 mg/kg CQ 1 h before cisplatin treatment and 24 and 48 h after treatment. This dosing regimen of CQ was chosen as it was shown to inhibit autophagy in the kidney of C57BL/6 wild-type mice by other groups (23). We observed expression of autophagy markers LC3 and p62 in the kidney 72 h after cisplatin treatment. Interestingly, basal autophagy levels in the kidney of nCDase^−/−^ mice seemed unaltered by CQ treatment. However, CQ inhibited cisplatin-induced autophagy in the kidney at this time point as evidenced by increased LC3-II and p62 expression (Fig. 6B–D). Increased expression of these markers occurs as CQ prevents autophagic flux by blocking autophagosome fusion with the lysosome (35).

### CQ treatment exacerbates cisplatin-induced AKI and apoptosis in nCDase^−/−^ mice

**Fig. 7:**
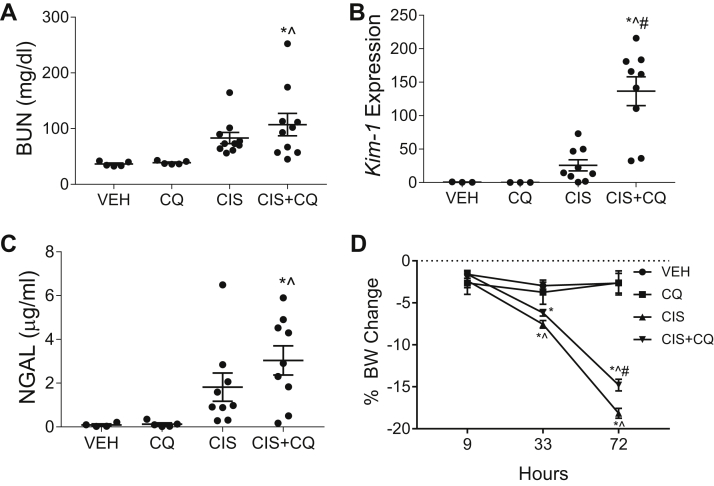
Chloroquine exacerbates cisplatin-induced AKI in neutral ceramidase knockout (nCDase^−/−^) mice. Mice were sacrificed 72 h following treatment. A: Blood urea nitrogen (BUN) was measured from plasma. B: Kidney injury molecule 1 (Kim-1) mRNA was measured by qRT-PCR from the renal cortex. C: Urinary NGAL was assessed. D: Percent body weight (BW) change from baseline was monitored each day after injection. VEH indicates vehicle-treated nCDase^−/−^ mice, CQ indicates chloroquine-treated nCDase^−/−^ mice, CIS indicates cisplatin-treated nCDase^−/−^ mice, and CIS+CQ indicates cisplatin and chloroquine–treated nCDase^−/−^ mice. Statistical differences were determined by a two-way ANOVA followed by a Tukey post-test. ∗Statistically different than vehicle-treated mice. #Statistically different than cisplatin-treated mice. ˆStatistically different than chloroquine-treated mice. Data expressed as mean ± SEM, n = 5–10.

nCDase^−/−^ mice treated with CQ and cisplatin had increased BUN and NGAL levels compared to nCDase^−/−^ mice treated with cisplatin alone (Fig. 7A, C). CQ treatment also led to a significant increase in mRNA expression of the AKI biomarker kidney injury molecule-1 in the kidney following cisplatin treatment (Fig. 7B). Body weight loss was less dramatic in cisplatin- and CQ-treated mice compared to cisplatin alone–treated mice, but both groups surpassed 10% body weight loss indicating overt toxicity (Fig. 7D).

**Fig. 8:**
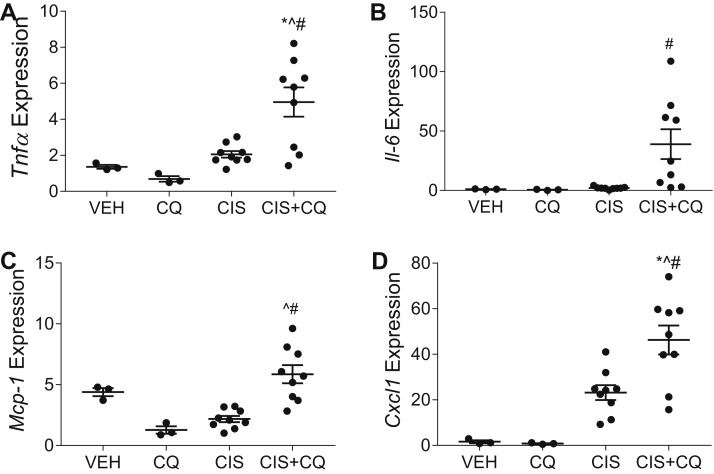
Chloroquine exacerbates inflammatory cytokine and chemokine production in neutral ceramidase knockout (nCDase^−/−^) mice. Mice were sacrificed 72 h following treatment. mRNA expression relative to control gene beta-2-microglobulin of (A) tumor necrosis factor α (*Tnfα*), (B) interleukin-6 (*Il-6*), (C) monocyte chemotactic protein-1 (*Mcp-1*), and (D) chemokine (C-X-C) ligand-1 (*Cxcl1*) were measured via real-time qRT-PCR. VEH indicates vehicle treated nCDase^−/−^ mice, CQ indicates chloroquine-treated nCDase^−/−^ mice, CIS indicates cisplatin-treated nCDase^−/−^ mice, and CIS+CQ indicates cisplatin and chloroquine–treated nCDase^−/−^ mice. Statistical differences were determined by a two-way ANOVA followed by a Tukey post-test. ∗Statistically different than vehicle-treated mice. #Statistically different than cisplatin-treated mice. ˆStatistically different than chloroquine-treated mice. Data expressed as mean ± SEM, n = 5–10.

Cisplatin-induced inflammatory cytokine production was also exacerbated by CQ treatment in the kidney cortex of nCDase^−/−^ mice. *Tnfα*, *Il-6*, *Mcp-1*, and *Cxcl1* mRNA expressions were all significantly elevated in the kidney of CQ and cisplatin-treated nCDase^−/−^ mice compared to cisplatin-treated nCDase^−/−^ mice (Fig. 8). These results indicate that CQ treatment in nCDase^−/−^ mice increases functional loss, kidney injury, and inflammatory cytokine production following cisplatin treatment.

**Fig. 9:**
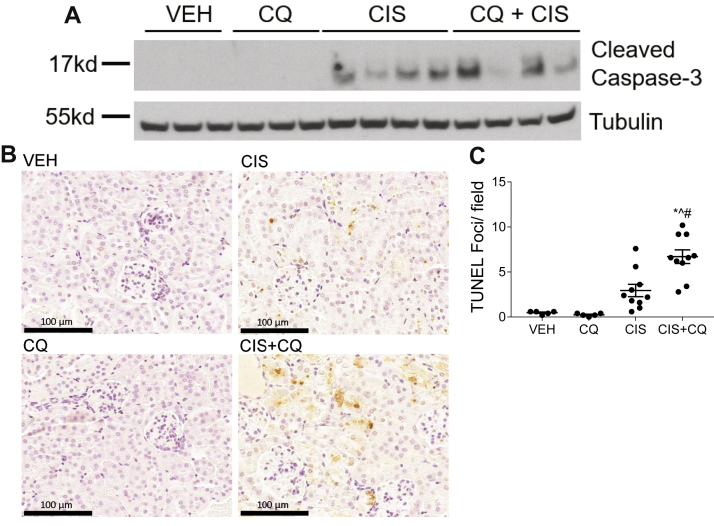
Chloroquine exacerbates cisplatin-induced apoptosis in neutral ceramidase knockout (nCDase^−/−^) mice. Mice were sacrificed 72 h following treatment. A: Western blot analysis was performed to assess relative protein levels of the indicated proteins in the renal cortex of mice. n = 3–4. The same protein lysates and loading control gene were used for Figure 6B. B, C: TUNEL assays were performed on paraffin-embedded kidney sections as an index of apoptosis. B: Representative images of TUNEL assays. C: TUNEL quantification. Statistical differences were measured by two-way ANOVA followed by Tukey post-test. Data expressed as mean ± SEM. n = 5–10. VEH indicates vehicle-treated nCDase^−/−^ mice, CQ indicated chloroquine-treated nCDase^−/−^ mice, CIS indicates cisplatin-treated nCDase^−/−^ mice, and CIS+CQ indicates cisplatin and chloroquine–treated nCDase^−/−^ mice. Statistical differences were determined by a two-way ANOVA followed by a Tukey post-test. ∗Statistically different than vehicle-treated mice. #Statistically different than cisplatin-treated mice. ˆStatistically different than chloroquine-treated mice. Data expressed as mean ± SEM, n = 5–10.

Apoptosis was examined in nCDase^−/−^ mice treated with CQ and cisplatin via CC3 expression and TUNEL staining. CC3 expression was elevated in nCDase^−/−^ mice treated with CQ and cisplatin compared to nCDase^−/−^ mice treated with cisplatin alone (Fig. 9A). Similarly, CQ treatment increased the number of TUNEL+ cells in nCDase^−/−^ mice following cisplatin treatment (Fig. 9B, C). These results suggest that CQ treatment exacerbates cisplatin-induced apoptosis in nCDase^−/−^ mice. Wild-type C57BL/6 mice were not used in this experiment as it was previously published that CQ treatment exacerbated cisplatin-induced AKI in these mice (23).

## Discussion

Sphingolipid metabolism is increasingly recognized as an important player in the development of kidney injury and disease (14). Our lab has demonstrated that manipulation of the generation and breakdown of ceramides can alter sensitivity to cisplatin-induced AKI (21). Additionally, we have shown that nCDase knockout protects mouse embryonic fibroblasts from nutrient and energy deprivation–induced cell death via upregulation of autophagic flux (22). Autophagy has also been shown to be protective from cisplatin-induced AKI (23). Therefore, we hypothesized that nCDase^−/−^ mice would be protected from cisplatin-induced AKI.

In this study, we demonstrate that nCDase^−/−^ mice have improved renal function, less kidney injury, and less tubule structural damage following 20 mg/kg cisplatin treatment compared to wild-type mice. Additionally, nCDase^−/−^ mice are protected from cisplatin-induced apoptosis and ER stress induction. We believe this protection is mediated by basally increased levels of autophagy in the kidney. LC3 expression was elevated in the kidneys of nCDase^−/−^ mice compared to wild-type mice. nCDase^−/−^ knockout in mouse embryonic fibroblasts was also previously shown to increase autophagic flux (22). Furthermore, CQ treatment sensitized nCDase^−/−^ mice to cisplatin-induced AKI. nCDase^−/−^ mice treated with CQ and cisplatin had worsened renal function, higher levels of kidney injury, and more apoptosis compared to nCDase^−/−^ mice treated with cisplatin alone.

We believe this sensitization is due to autophagy inhibition; however, it is important to note that CQ is known to affect cellular processes other than autophagy. CQ is a weak base and will therefore accumulate in any acidic cellular compartment. Lysosome morphology, Golgi organization, and endosomal trafficking were altered in several cell lines by CQ treatment (35). Additionally, CQ has been shown to affect membrane stability, signaling pathways, and immune activation (36, 37). We cannot definitively conclude that CQ is resensitizing nCDase^−/−^ mice to cisplatin-induced AKI via autophagy inhibition. Therefore, we also cannot conclude that nCDase^−/−^ knockout mediates protection from cisplatin-induced AKI via upregulation of autophagy. However, based on our previous in vitro work (22), we believe this is the primary mechanism of action, and increased autophagy is reducing cell death in the kidney following cisplatin treatment.

Ceramide generation has also been implicated in the regulation of cell death (15). Different forms of cellular stress, including cisplatin treatment, have been shown to induce ceramide accumulation and apoptosis in vitro (38, 39). Furthermore, inhibition of de novo ceramide synthesis prevented stress-induced apoptosis (38). As nCDase is responsible for the breakdown of ceramide into sphingosine and S1P, it may seem counter intuitive that nCDase inhibition would lead to decreased levels of cisplatin-induced apoptosis. However, inhibition of sphingolipid metabolizing enzymes does not always alter lipid levels in an expected way because of the complexity and interconnectedness of sphingolipid metabolic pathways. For example, loss of nCDase could be causing alterations in glycosphingolipid levels which have been shown to play a role in cisplatin-induced AKI (21). Additionally, sphingosine has also been shown to promote proximal tubule cell injury in vitro (40), suggesting that nCDase^−/−^ knockout could be protective by decreasing sphingosine levels in the kidney. Other studies have also demonstrated how nCDase loss can cause unexpected changes in the balance of ceramide, sphingosine, and S1P.

Snider *et al.* (41) demonstrated that nCDase^−/−^ mice had increased levels of S1P in colon epithelium compared to wild-type mice. These data suggest that nCDase^−/−^ mice could be protected by a counterintuitive increase in S1P and S1P receptor 1 (S1PR1) activation. S1PR1 activity has been shown to attenuate cisplatin-induced AKI by decreasing mitochondrial dysfunction and subsequent apoptosis (42). Additionally, the Okusa laboratory has shown a protective role for S1PR1 in both proximal tubule cells and endothelial cells in a model of renal ischemia/reperfusion-induced AKI (43, 44, 45, 46). However, studies show that activation of S1PR3 in dendritic cells is detrimental to renal ischemia/reperfusion-induced AKI (47, 48). It should be noted that it is difficult to assess how loss of nCDase is altering sphingosine and S1P levels in cisplatin-induced AKI as the effects may be cell type–specific and lipidomics analysis only allows us to assess steady state levels of whole tissue homogenates. Future studies employing in-depth lipidomics and MALDI imaging mass spectrometry (to get at the spatial changes in lipids) are needed to identify the lipid species involved. Additionally, future studies utilizing cell type–specific nCDase^−/−^ mice are important to better understand how loss of nCDase is attenuating cisplatin-induced AKI.

We hypothesize that nCDase^−/−^ knockout mediates protection from cisplatin-induced apoptosis and ER stress via upregulation of autophagy. Although the balance of ceramide, sphingosine, and S1P has been shown to affect levels of autophagy, the relationship is often determined by cell type and even subcellular localization of these lipids (49). Ceramide has been shown to induce autophagy (50, 51); however, S1P has also been shown to induce autophagy (52, 53). Interestingly, nCDase inhibition was also shown to increase autophagy in colon cancer cells (27). The molecular mechanisms by which nCDase regulates autophagic flux requires further investigation.

This study presents nCDase as a viable target for preventing cisplatin-induced AKI. We demonstrate that nCDase^−/−^ mice are protected from cisplatin-induced AKI as measured by decreased levels of apoptosis and ER stress. Interestingly, literature also supports nCDase inhibition as a strategy in treating colon cancer (27). Future studies should be aimed at identifying how nCDase inhibition may affect cancer treatment with cisplatin. It is also important to evaluate how nCDase inhibition alters long-term renal outcomes following cisplatin treatment, as cisplatin has been shown to induce renal fibrosis in mice given repeated low doses of cisplatin (30, 54). nCDase inhibition may play different roles in the biological processes occurring in chronic kidney injury (55). Taken together, these studies present nCDase inhibition as a novel target to prevent cisplatin nephrotoxicity and thereby improve cancer treatment.

## Data availability

All data described are contained within this manuscript.

## Conflict of interest

The authors declare that they have no conflicts of interest with the contents of this article.
